# Correction: Naked and necked gold nanoparticles prepared by submerged alternating current arc discharge in water

**DOI:** 10.1039/d2ra90135h

**Published:** 2023-01-12

**Authors:** K. Jankowski, J. Jabłońska, P. Uznański, S. Całuch, M. Szybowicz, R. Brzozowski, A. Ostafin, M. Kwaśny, M. Tomasik

**Affiliations:** a Institute of Nanotechnology and Nanobiology, Jacob of Paradies University Chopina St. 52, Bldg. 6 Gorzow Wielkopolski 66-400 Poland kjankowski@ajp.edu.pl; b Centre of Molecular and Macromolecular Studies, Polish Academy of Sciences Sienkiewicza 112 St. Lodz 90-363 Poland; c Faculty of Materials Engineering and Technical Physics, Poznan University of Technology Piotrowo 3A St. Poznan 61-138 Poland; d Institute of Optoelectronics, Military University of Technology Kaliskiego 2 St. Warsaw 00-908 Poland

## Abstract

Correction for ‘Necked gold nanoparticles prepared by submerged alternating current arc discharge in water’ by K. Jankowski *et al.*, *RSC Adv.*, 2022, **12**, 33955–33963, https://doi.org/10.1039/D2RA06050G.

The authors regret that the title shown in the original article was incorrect. The correct title is as shown above.

The Royal Society of Chemistry apologises for these errors and any consequent inconvenience to authors and readers.

## Supplementary Material

